# Assessing Callous-Unemotional Traits Across Early Adolescence: Further Evaluation of Short Versions

**DOI:** 10.1007/s10578-024-01746-7

**Published:** 2024-09-06

**Authors:** Giuseppe Corbelli, Valentina Levantini, Pietro Muratori, Vincenzo Paolo Senese, Carmela Bravaccio, Simone Pisano, Gennaro Catone, Marinella Paciello

**Affiliations:** 1 Faculty of Psychology, International Telematic University Uninettuno, Rome, Italy; 2 Department of Languages and Literatures, Communication, Education and Society, University of Udine, Udine, Italy; 3 Scientific Institute of Child Neurology and Psychiatry, IRCCS Fondazione Stella Maris, Calambrone, Pisa, Italy; 4 Psychometric Laboratory, Department of Psychology, University of Campania Luigi Vanvitelli, Caserta, Italy; 5 Department of Translational Medical Sciences, Federico II University, Naples, Italy; 6 Department of Educational, Psychological and Communication Sciences, Suor Orsola Benincasa University, Naples, Italy

**Keywords:** Adolescence, Inventory of Callous-Unemotional Traits, Longitudinal, Strengths and Difficulties Questionnaire, Validity

## Abstract

Literature on the Inventory of Callous-Unemotional (ICU) traits has suggested different versions of the instrument for assessing these traits during development. However, consensus on the instrument version and the best factorial solution remains a matter of debate, with only a few studies having validated ICU versions from a longitudinal perspective. The current study aims to contribute to the literature by comparing ICU models in a longitudinal sample of early adolescents (N = 739; 70.6% of eligible subjects, 371 females and 368 males, in the 6th grade at baseline assessment and in the 8th grade at the second assessment). We tested the validity of various versions of the ICU scales and their respective dimensions by conducting a series of confirmatory factor analyses to verify the factor structure, alongside assessments of internal consistency. For the best-fitting structure, we then analyzed gender and longitudinal invariance in addition to construct and predictive validity, using internalizing and externalizing criteria as well as prosocial behavior. From the comparative analysis, it emerged that the abbreviated 11-item ICU scale version displayed overall better data fit than the full 24-item version. Moreover, its confirmed gender invariance underscores its applicability across genders within the studied age group. With regard to longitudinal invariance, our findings advise caution when comparing ICU scores across early adolescence. Practical implications are discussed.

## Introduction

Scientific literature has extensively highlighted that callous-unemotional (CU) traits (i.e., lack of guilt and remorse, shallow affect, and reduced empathy) [1] are related to more persistent and severe aggressive and antisocial behavior, long-term adverse outcomes [2–5], as well as poorer responses to psychosocial and pharmacological treatments [6–8]. The consensus on the strong connection between CU traits and the emergence of conduct problems in children and adolescents [9] has led to the development of measures apt to assess this relevant construct [10]. Among the instruments, the Inventory of Callous Unemotional Traits (ICU) [11] is the only one entirely focused on CU traits. The original version of ICU is a 24-item, multi-informant (parent-, teacher-, self-report) tool that comprehensively measures youths' CU traits, and its items were used to define the symptoms of the Limited Prosocial Emotion (LPE) specifier for Conduct Disorder [1]. Moreover, the ICU has been translated into several languages, and a wealth of studies provided evidence of its validity (for a meta-analysis, see [12]).

However, subsequent studies have shown different factorial solutions and proposed different shortened versions during the screening and monitoring phases of CU traits in development (e.g., [13–15]). The consensus on the instrument version and the best factorial solution is still a matter of debate, and only a few studies have validated ICU versions adopting a longitudinal perspective [16]. In the present study, we aim to contribute to the literature by first assessing the factorial structures of various version of ICU scales in a longitudinal sample of non-clinical early adolescents. Following this comparative analysis of scale versions to identify the best fitting one, we then examine gender and longitudinal invariance and assess the construct validity of the selected ICU dimensions over a two-year period. Interest in studying CU traits development has been growing in the literature, and longitudinal studies [17, 18] have shown high variability both at the inter-individual level and at the individual level. Further investigation in this regard could assist scholars and clinicians in making more targeted choices regarding the instrument version to use for assessing CU traits during early adolescent development.

### ICU: Dimensions and Measures

The ICU was initially conceptualized as a unidimensional measure aimed at capturing the affective dimension of psychopathy. Consistently, different studies have shown that the total ICU score validly designate a peculiar subgroup of children and adolescents with aggressive and antisocial behavior, showing acceptable internal reliability and robust associations with constructs typically associated with psychopathic traits, e.g., higher externalizing problems, delinquency, and proactive aggression, poorer empathic skills [19–21]. Though less investigated, some evidence has shown an association between ICU scores and internalizing problems (e.g., [22]), which appear to characterize, at least, a subgroup of children with high levels of CU traits with particularly severe outcomes (e.g., higher conduct problems, lower self-esteem) [23, 24].

However, a wealth of subsequent studies proposed a three-factor bifactor structure with a general CU factor and three subfactors (i.e., callous, uncaring, unemotional) as the best-fitting model for the ICU (e.g., [11, 15, 22]). The three independent factors should reflect the main characteristics of youth with high CU traits, specifically, their lack of remorse and empathy (callousness factor), their uncaring attitude about others' feelings and performance in relevant activity (uncaring factor) and, lastly, their shallow and deficient emotional expression and experience (unemotional factor). Although frequently considered the best factor structure, the bifactor model's fit statistics are usually highly variable and unsatisfactory. Indeed, multiple studies conducted in several countries with different samples (e.g., middle school students, at-risk adolescents, and juvenile offenders) were not able to replicate this model [25–28], questioning its actual generalizability. Furthermore, some authors have also raised concerns about the validity of the Unemotional subscale and wondered whether it should be retained or removed from the ICU [11]. In this regard, the Unemotional subscale score has frequently shown unexpected/inconsistent correlations with constructs relevant to CU traits (e.g., antisocial behavior [22, 29]) and poorer relationships with the overarching CU traits construct (e.g., [11, 25]),in a recent network analysis, unemotional items were located in a peripheral position, with fewer links apparent to callousness and uncaring items [30]. It has been suggested that the Unemotional subscale may better represent mixed emotional processes poorly related to antisocial behavior or psychopathic traits. These items might be interpreted as describing a general tendency to hide emotions [14] or being shy, withdrawn, or anhedonic [12].

Concerns regarding the factorial validity of the ICU led to the development of refined and shortened versions of the scale. These versions showed improved fit compared to the original 24-item bifactor model across multiple studies. For instance, Houghton et al. [15] considered, in a sample of 268 children aged 12–13 years, a 16-item self-report ICU version, which included only items from two factors, namely Callousness and Uncaring. Subsequently, Hawes et al. [14] proposed a 12-item version of the parent-report ICU in a sample of 6 to 12 years old boys with conduct problems, consisting of Callousness and Uncaring dimensions. This version excluded all the items originally included in the Unemotional subscale but one (item 6: “Does not show emotions”), which was loaded into the Callousness factor. Colins et al. [13] also tested an 11-item version of the self-report ICU form without item 6 in a sample of detained adolescents, which showed better fit statistics. Through item-response theory, Ray et al. [21] proposed a 10-item version of the self-reported ICU in a large sample of first-time justice-involved adolescents. Finally, Gao and Zhang [31] tested in a community sample of children (8–10 years old) a 13-item version of the parent- and child-report, formed by two factors, i.e., Callousness and Uncaring.

The models most extensively tested and supported are those proposed by Hawes et al. [14] and Colins et al. [13], as evidenced by subsequent studies including Paiva-Salisbury et al. [32] and Waller et al. [33], which compared different shortened versions of the ICU. Wang et al. [16] examined the factor structure of the shortened versions of the ICU with data from multiple informants in a sample of community Chinese children. All the short versions reported good fit statistics, with the Colins et al. model deemed the best. This study also provided evidence of cross-informants and longitudinal invariance of this version of the ICU. Similar results were reported by Allen et al. [34]: findings showed that the original ICU was a poor fit in the study sample, and that 11-item ICU provided the best fit in a sample of UK children (aged 11–14 years) and a sample of Chinese children (aged 10–13 years). The authors also provided evidence of gender invariance of the 11-item ICU in both samples.

### Present Study

The current study aims to contribute to the above-mentioned literature by comparing the ICU models in a sample of early adolescents. Specifically, our objectives include:

- testing the validity of different versions of ICU scales and their respective dimensions;
- verifying the stability of the scale across both genders and across time;
- validating the scales while concurrently considering adaptation dimensions and prospectively assessing their impact.

To these aims, we conducted a series of confirmatory factor analyses (CFA) to compare the different models described above, explore the internal consistency of the best-fitting structure, and test its gender and longitudinal invariance. We specifically tested the gender differences in the ICU scores and the associations between the ICU scores, children's emotional and behavioral difficulties, and prosocial behavior cross-sectionally and longitudinally.

Based on extant literature, we expect that the 11 or 12-item version of the ICU would better fit our data (e.g., [16]) and to be invariant across genders (e.g., [34–36]) and longitudinally [16]. We hypothesized that males would report higher scores than females on the ICU total and subscales scores [25, 37, 38]. We also expect that the ICU scores would be associated with more externalizing and internalizing symptoms and reduced prosocial behavior [4, 5, 31, 39, 40].

## Methods

### Sample and Procedure

This study is part of a large longitudinal, school-based project, the Bullying and Youth Mental Health Naples Study (BYMHNS, see: [41]). Data collection was obtained throughout the administration of self-report scales to participants. Twelve middle schools (with a total population of approximately 4445 students) joined the project, from the metropolitan and suburban area of Naples, Italy, to ensure representativeness (geographical criterion). The first wave of assessment (T1) took place in the school year 2015/2016, featuring the complete ICU-24 scale alongside the Strengths and Difficulties Questionnaire. The subsequent wave (T2) was conducted in the 2017/2018 school year, with a new measurement of the Strengths and Difficulties Questionnaire. In the first wave, a total sample of 2959 students, which represented the 66.6% of the total number of subjects that attended the schools at that period, was recruited, in the second wave, those attending the first grade during the first wave (attending the third grade during the second wave) were re-assessed. On a total number of 1048 subjects, 868 of them were re-assessed, representing 82.8% of eligible subjects. After matching T1 with T2 data and excluding some subjects with invalid data, a total sample of 739 subjects with full and valid data was achieved (70.6% of eligible subjects, 371 females and 368 males, 6th grade at baseline assessment, 8th grade at second assessment).

An attrition analysis comparing demographic and general psychopathology baseline variables revealed that over and above sex and age, those lost at T2 presented more externalizing symptoms at T1 (OR = 1.073, p = 0.005), although the difference is marginal.

The ethical committee of the Campania University Luigi Vanvitelli approved the study (N. 500, 29/04/2016). Parents gave their written informed consent and participants their assent. This study was performed in line with the principles of the Declaration of Helsinki.

### Measures

#### Inventory of Callous Unemotional Traits

The Inventory of Callous-Unemotional Traits (ICU; [11, 25]) is a 24-item, self-report scale developed to assess four aspects of CU traits: Callous, Lack of Remorse, Lack of Concern about Performance and Unemotionality. Twelve positively and 12 negatively keyed items are rated on a 4-point ordinal scale ranging from 0 to 3. A more detailed description of the ICU and its structure is provided in the introduction.

#### Strengths and Difficulties Questionnaire

The Strengths and Difficulties Questionnaire (SDQ) [42–44] is a 25-item questionnaire assessing emotional and behavioral difficulties and strengths in children and adolescents. Answers are provided on a 3-point Likert scale. The SDQ involves five subscales, namely Hyperactivity-Inattention, Emotional Symptoms, Conduct Problems, Peer Problems, and Prosocial Behavior. Subscales’ scores can be combined to get an Internalizing Problems (Emotional Symptoms + Peer Problems) and an Externalizing Problems (Hyperactivity + Conduct Problems) score.

### Data Analysis

#### Comparative Choice of the Best-Fitting ICU Version

Preliminarily, multivariate outliers were identified. By calculating each observation's Mahalanobis distance and comparing it to the critical value from the chi-squared distribution at a 0.001 alpha level, data points with Mahalanobis distances exceeding this critical chi-squared value were considered outliers, and were thus excluded from the dataset [45]. Hence, to verify the assumption of multivariate normality, Mardia's test was applied [46]. Internal consistency indices for the ICU scales were compared, including Cronbach’s alpha [47], and total composite reliability [48]. Cronbach’s alpha, though a widely recognized measure of internal consistency, not only depends on the assumption of tau-equivalence but also, due to its sensitivity to item number, can sometimes provide a skewed view of a scale’s reliability, particularly in shorter scales like the one under investigation here. Therefore, as these scale dimensions can be theoretically collapsed into a single composite ICU index, total composite reliability could provide a more general and robust estimation of internal consistency.

Through confirmatory factor analysis (CFA), we assessed and compared the fit of five of the most clinically-relevant brief ICU versions as delineated by Wang et al. [16] and Allen et al. [34], as well as the complete 24-item ICU scale. Following Kline [49] and Hu and Bentler [50], both the acceptability of each model and the choice of the comparatively best-fitting model were assessed through the chi-square model test statistic, alongside with its degrees of freedom, the Bentler Comparative Fit Index (CFI) ranging from 0 to 1 (0.90 indicating an acceptable fit, and 0.95 a good fit), the Tucker–Lewis Index (TLI) with a minimum value of 0 (0.90 indicating an acceptable fit, and 0.95 a good fit), the Root Mean Square Error of Approximation (RMSEA) and its 90% Confidence Interval (ranging from 0 to 1, with 0.08 indicating an acceptable fit, and 0.05 a good fit), the Standardized Root Mean square Residual (SRMR), that for a perfect model equals 0 (0.10 indicates an acceptable fit, and 0.08 a good fit).

#### Gender Measurement Invariance

To confirm whether the construct can be meaningfully compared across different genders, measurement invariance for the selected version of the Inventory of Callous-Unemotional Traits was assessed in a structural equation modeling framework through confirmatory factor analysis (CFA). To do that, we executed a hierarchical sequence of tests across gender groups to determine the significance of differences in model fit under increasingly restrictive equality constraints [51–53]. Initially, configural invariance was evaluated to ensure the factor structure of the model was consistent between gender groups. This served as a foundation for further testing metric (or weak factorial) invariance, where we investigated the equivalence of factor loadings across genders. Subsequent analyses tested for scalar (or strong factorial) invariance by constraining both the factor loadings and intercepts to equality between genders. The final and most stringent level of invariance assessed was strict factorial invariance, which also included the equality of residual variances across gender groups. We considered achieving at least the first three levels of invariance (configural, metric, and scalar) as indicative of the instrument's ability to make valid comparisons between groups or occasions [54, 55].

#### Longitudinal Measurement Invariance

To ensure that any observed longitudinal differences in means are attributable to changes in the evaluated construct rather than to alterations in the measurement model over time, we assessed longitudinal invariance for the selected instrument. Employing the hierarchical testing procedure outlined previously, we began with configural invariance to verify the stability of the factor structure between T1 and T2. Successive tests for metric and scalar invariance were conducted to assess the equality of factor loadings and intercepts, respectively. Finally, strict invariance was considered, which tests for the equality of residual variances across the two time points.

#### Construct and Predictive Validity of ICU Dimensions

The relationship between the previously selected ICU scale dimensions and those of the Strengths and Difficulties Questionnaire (SDQ) both at T1 and T2 was examined with the aim of exploring construct validity and the predictive value of ICU scale through the relations with theoretically related traits and symptoms [56]. First, concurrent partial correlations of the prosocial, externalizing, and internalizing problems scales of the SDQ with the ICU factors were analyzed. Then, linear regression models were fitted to assess the predictive validity of the ICU scale measured at T1 on the SDQ dimensions at T2, after controlling for the corresponding values at T1. All analyses were conducted within the R environment v.4.0.4. [57].

## Results

### Comparative Choice of the Best-Fitting ICU Version

From the initial dataset, 61 data points were excluded because they were identified as multivariate outliers; the final total sample, therefore, consists of 730 observations. Mardia's test for multivariate normality showed a statistically significant departure from multivariate normality for both skewness (8876.66, p < 0.001) and kurtosis (35.11, p < 0.001), and the same results were found consistently in T2 observations (skewness: 6728.02, p < 0.001; kurtosis: 30.11, p < 0.001). Little's test was significant at the alpha threshold of 0.05, thus leading to the rejection of the null hypothesis that the missing data are produced by an MCAR mechanism. Nevertheless, on the basis of the borderline result of the test (*χ*2[892] = 965.0, p = 0.04) and attrition analysis, this is considered sufficient to assume that the missing data are at least MAR, allowing the application of the maximum likelihood information method (FIML) for handling missing data at T2 without producing significantly biased parameter estimates and standard errors [58]. The analysis of the internal consistency across six versions of the Inventory of Callous-Unemotional Traits (ICU) revealed varied levels of reliability for different scales (see Table 1). In line with prior research findings [56], Cronbach's alpha values mostly fell within the lower bound of acceptability. When considering the factorial structure of the instruments, composite reliability indices approached the acceptability threshold for all scales.

**Table 1:** Internal consistency indices for the six considered ICU versions

ICU model	Cronbach’s α	Composite reliability
ICU-24	0.72	0.73
ICU-16	0.71	0.69
ICU-13	0.64	0.69
ICU-12	0.62	0.69
ICU-11	0.62	0.69
ICU-10	0.71	0.68

To account for the multivariate non-normal distribution of the observed variables, the models were tested with CFA by maximum likelihood estimation with robust standard errors (Huber-White) and a test statistic equivalent to that of the Yuan-Bentler test, suitable for both complete and incomplete data. All the declared fit indices for the scales investigated are presented (and compared) in Table 2. Not for all given scales did the fit indices indicate satisfactory alignment of empirical results with the presumed measurement model: the full 24-item scale (ICU-24, 3 factors) did not reach acceptable fit thresholds, and the same can be said for the 16-item version (ICU-16, 2-factor) despite showing better indices. The 12- and 13-item versions, on the other hand, showed overall acceptable fit indices. The 11-item scale (ICU-11, 2 factors) achieved the best CFA results overall with CFI and TLI well above the good fit threshold, and the lowest RMSEA and SRMR, further indicating an excellent fit. Conversely, the 10-item version (ICU-10, 1 factor) showed again unacceptable fit indices. Therefore, the absolute and comparative evaluation of the different ICU scale variants led us to select the ICU-11 model proposed by Colins and colleagues as the best-fitting version.

**Table 2:** Confirmatory factor analysis (fit indices) for the six considered versions of the Inventory of Callous-Unemotional traits (T1)

Model	yb-χ^2^	df	CFI	TLI	RMSEA [90% CI]	SRMR
ICU-24 (3 Fs^a^)	923.41	249	0.72	0.69	0.06 [0.06, 0.07]	0.08
ICU-16 (Houghton, 2 Fs)	396.29	103	0.81	0.78	0.07 [0.06, 0.07]	0.07
ICU-13 (Gao & Zhang, 2 Fs)	124.97	64	0.93	0.92	0.04 [0.03, 0.05]	0.04
ICU-12 (Hawes, 2 Fs)	105.28	53	0.94	0.93	0.04 [0.03, 0.05]	0.04
ICU-11 (Colins, 2 Fs)	68.70	43	0.97	0.96	0.03 [0.01, 0.04]	0.03
ICU-10 (Ray, 1 F)	215.56	35	0.85	0.80	0.09 [0.08, 0.10]	0.05

^a^Number of considered factor(s)

All p-values for chi-square tests are < .01

Item 12 presented a low, albeit significant, standardized factor loading (0.19, p < 0.001), with all other loadings exceeding the suggested threshold of 0.40 [59], ranging from 0.30 to 0.72. Consequently, in accordance with the findings reported by Wang et al. [60], the 11-item version of the ICU was chosen as the most suitable instrument, balancing model parsimony with goodness of fit. The two factors were significantly correlated (r = .29, p < 0.001). Table 3 shows the factor loadings for each item of the ICU-11.

**Table 3:** ICU-11 factor loadings of the 2-factor solution

Factor	Item	Unstandardized factor loading	SE	Standardized factor loading	R2
Callousness	Item 4	0.276	0.040	0.400	0.160
	Item 9	0.387	0.057	0.428	0.183
	Item 11	0.417	0.054	0.448	0.201
	Item 12	0.145	0.042	0.193	0.037
	Item 18	0.438	0.047	0.546	0.298
	Item 21	0.303	0.052	0.351	0.123
Uncaring	Item 5	0.595	0.040	0.626	0.392
	Item 8	0.507	0.040	0.487	0.237
	Item 16	0.574	0.037	0.721	0.520
	Item 17	0.633	0.042	0.660	0.435
	Item 24	0.289	0.044	0.302	0.091

All factor loadings are statistically significant (p ≤ .001))

### Gender Measurement Invariance

The results of the gender invariance analyses are presented in Table 4. For the likelihood ratio test, the Yuan–Bentler scaled chi-square difference was scaled by the difference in degrees of freedom and the scaling correction factors for the considered models [61, 62]. All indices indicate an adequately good fit. Along with chi-square differences, also the differences in Comparative Fit Index (CFI) between the configural and the metric invariance models (ΔCFI = 0.005) and between the metric and scalar invariance models (ΔCFI = 0.007) are below the threshold of 0.01 suggested by Cheung and Rensvold [63],the difference between scalar and strict invariance models (ΔCFI = 0.012) remains reasonably close to this limit as well. Thus, these results indicate that the ICU-11 is meaningfully measuring the same construct across genders in the considered sample.

**Table 4:** Results of gender measurement invariance

Model invariance	yb-χ^2^ (df)	CFI	TLI	RMSEA [90% CI]	SRMR	Δχ^2^ (Δdf)	p	Decision
Configural	110.37 (86)	0.97	0.96	0.03 [0.00, 0.04]	0.04	–	–	–
Metric	113.72 (95)	0.98	0.97	0.02 [0.00, 0.04]	0.04	4.90 (9)	0.84	Accept
Scalar	128.33 (104)	0.97	0.97	0.03 [0.00, 0.04]	0.05	14.97 (9)	0.09	Accept
Strict	129.15 (115)	0.98	0.98	0.02 [0.00, 0.04]	0.05	6.18 (11)	0.86	Accept

N = 730; N_male_ = 363; N_female_ = 367

Building upon the established measurement invariance, the scores of the two factors of the ICU-11 between males and females were compared. Levene’s Test indicated a violation of the homogeneity of variances assumption for both the Callous factor, F(1, 728) = 4.94, p = 0.027, and the Uncaring factor, F(1, 728) = 4.13, p = 0.042. Consequently, Welch's t-tests, which do not require equal variances, were conducted to compare the means. For the Callous factor, a significant difference was found in scores between males (M = 2.92, SD = 2.91) and females (M = 2.32, SD = 2.43): t(703.36) = 3.01, p = 0.003, d = 0.22. Similarly, for the Uncaring factor, a significant difference was observed, with males (M = 4.85, SD = 3.28) scoring higher than females (M = 3.96, SD = 2.93): t(717.01) = 3.88, p < 0.001, d = 0.29.

### Longitudinal Measurement Invariance

Regarding longitudinal invariance analysis for the ICU-11 across T1 and T2, the configural invariance model presented a very good fit (see Table 5), suggesting a stable factor structure over time. Metric invariance was similarly upheld, confirming that the relationship between each item and the underlying construct remains consistent across the two time points. The difference in Comparative Fit Index (CFI) between the configural and metric invariance models is 0.002, which is again below the cutoff value suggested by Cheung and Rensvold [63]. However, the Chi-square difference between the metric and scalar invariance models is relatively large and statistically significant, and also the ΔCFI of 0.051 exceeds the threshold of 0.01. Consequently, the more restrictive scalar invariance model—which equates intercepts across time points—does not adequately explain the empirical data obtained for the considered sample.

**Table 5:** Results of longitudinal measurement invariance

Model invariance	yb-χ^2^ (df)	CFI	TLI	RMSEA [90% CI]	SRMR	Δχ^2^ (Δdf)	p	Decision
Configural	164.39 (86)	0.96	0.95	0.04 [0.03, 0.05]	0.04	–	–	–
Metric	177.33 (95)	0.96	0.95	0.04 [0.03, 0.05]	0.04	13.08 (9)	0.16	Accept
Scalar	285.61 (104)	0.91	0.90	0.05 [0.05, 0.06]	0.05	121.27 (9)	< .001	Reject

N_T1_ = 730; N_T2_ = 722

### Construct and Predictive Validity of ICU Dimensions

To assess construct validity, correlations between the ICU-11 and SDQ factors at both time points were examined (see Table 6). The correlations were adjusted for the other component of the ICU-11, ensuring that each association reflected the unique contribution of each ICU-11 factor.

**Table 6:** Concurrent partial correlations between ICU-11 and SDQ dimensions at T1 and T2

		EPS	CPS	HYP	PEP	PRO	INT	EXT
T1	Callousness	0.16***	0.30***	0.22***	0.15***	− 0.05	0.18***	0.29***
	Uncaring	− 0.13***	0.13***	0.16***	0.15***	− 0.51***	− 0.02	0.17***
T2	Callousness	0.09*	0.37***	0.29***	0.14***	− 0.18***	0.12***	0.37***
	Uncaring	− 0.20***	0.12**	0.15***	0.07	− 0.53***	− 0.10*	0.16***

The correlations of each ICU dimension with the SDQ indicators are controlled for the effect of the other dimension

*EPS* emotional symptoms, *CPS* conduct problems, *HYP* hyperactivity, *PEP* Peer relationship problems, *PRO* prosocial behavior, *INT* internalizing problems, *EXT* externalizing problems

*p ≤ .05, ** p ≤ .01, *** p ≤ .001

Initially, the Callousness factor displayed a positive correlation with externalizing problems at T1 (r = 0.29, p < 0.001), which increased at the second time point (r = 0.37, p < 0.001). Examining each dimension individually, the correlation of the Callousness trait with conduct problems alone was positive at T1 (r = 0.30, p < 0.001) and increased at T2 (r = 0.37, p < 0.001). Its correlation with hyperactivity was weakly positive at the first time point (r = 0.22, p < 0.001) and increased at the second (r = 0.29, p < 0.001). Overall, these changes suggest a growing association between the Callousness factor and externalizing behaviors measured across the two time points with ICU-11. Regarding internalizing problems, the ICU-11 Callousness factor's correlation was weak at T1 (r = 0.18, p < 0.001) and became weaker at T2 (r = 0.12, p = 0.001). Considering the components of internalizing problems separately, the Callousness factor showed a weak positive correlation with peer relationship problems at both the first (r = 0.15, p < 0.001) and second time points (r = 0.14, p < 0.001). In contrast, its correlation with emotional symptoms was weak at T1 (r = 0.16, p < 0.001) and further reduced at T2 (r = 0.09, p = 0.019). Moreover, the Uncaring factor's correlations with externalizing problems were weak at both T1 (r = 0.17, p < 0.001) and T2 (r = 0.16, p < 0.001). The correlations with the individual components of externalizing problems remained relatively weak across both time points, at r = 0.13 (p < 0.001) and r = 0.12 (p = 0.001) for conduct problems, and r = 0.16 (p < 0.001) and r = 0.15 (p < 0.001) for hyperactivity, respectively. The Uncaring factor's correlation with internalizing problems was negligible at T1 (r = − 0.02, p = 0.668), but became significant at the second time point (r = − 0.10, p = 0.008). This ICU-11 factor exhibited a weak yet significant negative correlation with emotional symptoms at both T1 (r = − 0.13, p < 0.001) and T2 (r = − 0.20, p < 0.001), and a comparable but positive correlation with peer relationship problems at the first time point (r = 0.15, p < 0.001) which becomes non-significant at the second time point (r = 0.07, p = 0.057). In terms of prosocial behavior, the Callousness factor did not show a significant correlation at T1 (r = − 0.05, p = 0.156), but the same becomes significantly negative at T2 (r = − 0.18, p < 0.001). Conversely, the Uncaring factor demonstrated a moderate negative correlation with prosocial behavior at both T1 (r = − 0.51, p < 0.001) and T2 (r = − 0.53, p < 0.001).

Linear regression analyses were conducted to evaluate the predictive power of the Callousness and Uncaring factors at T1 for the various SDQ dimensions at T2, with each model adjusted for the corresponding T1 measurement of the SDQ outcome. Overall, the results for externalizing problems show that the two ICU factors are not significant predictors (Callousness (T1): β = 0.04, t(686) = 0.99, p = 0.323; Uncaring (T1): β < 0.01, t(686) = − 0.01, p = 0.992; Externalizing problems (T1): β = 0.46, t(686) = 12.93, p < 0.001). For conduct problems, it can be seen how among the ICU factors only Callousness shows a small but significant positive influence (Callousness (T1): β = 0.08, t(703) = 2.09, p = 0.037; Uncaring (T1): β = 0.01, t(703) = 0.24, p = 0.808; Conduct problems (T1): β = 0.31, t(703) = 8.26, p < 0.001). The results for hyperactivity show that neither ICU factor significantly influences the outcome (Callousness (T1): β = 0.04, t(703) = 1.11, p = 0.267; Uncaring (T1): β = 0.02, t(703) = 0.58, p = 0.564; Hyperactivity (T1): β = 0.43, t(703) = 12.30, p < 0.001). The results for internalizing problems show that the ICU factors exert no significant effect (Callousness (T1): β = − 0.07, t(703) = − 1.91, p = 0.057; Uncaring (T1): β = − 0.05, t(703) = − 1.53, p = 0.126; Internalizing problems (T1): β = 0.48, t(703) = 14.09, p < 0.001). In detail, the results for peer relationship problems show that both Callousness and Uncaring at T1 do not significantly impact this SDQ factor measured at the second time point (Callousness (T1): β < 0.01, t(703) = 0.01, p = 0.992; Uncaring (T1): β = − 0.01, t(703) = − 0.19, p = 0.851; Peer relationship problems (T1): β = 0.38, t(703) = 10.47, p < 0.001). Meanwhile, for emotional symptoms the two ICU factors are both statistically significant as negative predictors, while showing a small influence (Callousness (T1): β = − 0.07, t(703) = − 1.96, p = 0.050; Uncaring (T1): β = − 0.07, t(703) = − 2.08, p = 0.038; Emotional symptoms (T1): β = 0.42, t(703) = 12.04, p < 0.001). Finally, for prosocial behavior, the analysis shows significant negative effects from both ICU factors (Callousness (T1): β = − 0.07, t(703) = − 2.01, p = 0.045; Uncaring (T1): β = − 0.13, t(703) = -3.11, p = 0.002; Prosocial behavior (T1): β = 0.22, t(703) = 5.23, p < 0.001).

## Discussion

From the comparative analysis, it emerges that the 11-items versions of the ICU scale showed overall better data fit than the full-length version, corroborating findings by Colins et al. [13], Wang et al. [16] and Allen et al. [34]. Notably, the ICU-24 scale displayed the poorest fit for the sample, whereas the ICU-11 version achieved the best fit. Furthermore, in line with the literature, the reduced 11-item scale captures the two dimensions of callousness and uncaring, while excluding the unemotional subscale factor, which has frequently shown inconsistent findings. (e.g., antisocial behavior [22, 29]). Indeed, considering the widespread application and significance of this scale in both research and clinical settings, the adoption of a shorter scale not only could enhance its overall measurement properties but also simplifies administration, particularly to adolescent populations. The brevity of the scale could facilitate more efficient and less burdensome assessment processes, thereby increasing its usefulness in diverse settings. Moreover, the results demonstrate that the ICU-11 items version is reliable, valid and invariant for both male and female adolescents. Its confirmed gender invariance underscores its applicability across genders within the studied age group. This robust metric structure ensures that cross-gender comparisons are valid and meaningful, also in view of the fact that both literature and present findings indicate that both traits of callousness and uncaring are more pronounced among males.

With regard to longitudinal invariance, our findings advise caution when comparing ICU scores across early adolescence. The affirmation of both configural and metric invariance across the two time points supports the notion that the scale's two-factor structure comprising callousness and uncaring traits persists throughout adolescence. This consistency suggests that each item remains a valid indicator of its respective construct across the age spectrum examined. These findings suggest that the relative significance attributed to the scale's items in relation to the callous and uncaring dimensions remains stable over time, enabling valid interpretation of these traits among individuals aged 12–14 years. However, the inability to establish scalar invariance raises caution against potential overinterpretations of the findings. Specifically, it complicates the assessment of longitudinal mean changes, as it impedes direct comparisons of raw scores over the considered time span. This limitation is due to the consequent difficulty in ensuring that observed variations in mean scores of the callousness and uncaring dimensions, as measured by this specific instrument, reflect actual changes in the underlying constructs rather than measurement artifacts. For instance, such discrepancies could be an effect of the different life experiences (e.g., social and peer challenges, increased academic demands) encountered by individuals at ages 12 and 14, including their exposure to situations relevant to the scale's items. These experiences might influence the extent of their agreement with the items' content, without altering their overall comprehension of the items or the items' interrelations; this could account for the observed consistency in the scale's configural and metric properties over time. In other words, the variation in response intercepts may not accurately represent shifts or changes in the callous and uncaring factors, but rather indicate a different interpretation of the item content attributed to developmental maturation and more diverse life experiences. However, this finding cannot be compared with earlier studies because their time points are different, as they don’t take into account the transition into adolescence.

Nonetheless, because the establishment of metric invariance confirms that the measurement of the construct and the integrity of its factor structure remain consistent over time, it becomes feasible to examine the relationships between dimensions and variables in relation to the two identified factors, callousness and uncaring, across the two different occasions. These relations can therefore be rightfully considered and discussed, and interpreting scores from these scale factors in correlation and regression analyses against clinically significant outcomes could also be justified, with the magnitude of the findings being comparable across the two time points.

In our case, regarding the psychological and behavioral correlates of ICU dimensions, interesting findings emerge both concurrently and longitudinally.

Concerning the cross-sectional correlations, it is possible to appreciate the differential role of the Callousness and Uncaring factors on target SDQ dimensions. Indeed, these ICU dimensions differ in their relationship with conduct disorders (which is stronger for the Callousness factor), with internalizing problems (which correlate only with Callousness), and prosocial behavior, which correlates only with the uncaring factor. Moreover, their predictive value becomes even more evident when observing their longitudinal impact on SDQ dimensions by controlling for the stability of the outcome. In this case, while both Callousness and Uncaring factors have a similar impact on emotional symptoms, their contributions differ with respect to conduct problems and prosocial behaviors. Specifically, for conduct problems, Callousness remains a significant dimension, whereas the contribution of Uncaring is not significant. Conversely, for prosocial behaviors, though both ICU factors are significant, Uncaring assumes a more crucial predictive role. For the other dimensions, no significant contributions were found when controlling for the stability of the SDQ dimension.

In sum, concerning externalizing behavioral problems, both dimensions are positively correlated, consistent with existing literature [19–21]. However, it is noteworthy that callousness exhibits a stronger association over time. Contrary to this, negative associations with prosocial behavior are observed, with Uncaring playing a predominant role. This outcome is expected, as prosocial behavior is inherently a behavioral expression of caring. Finally, results showed that the callousness scale—but not the uncaring one—was marginally associated with internalizing symptoms. Likewise, Essau et al. [22] found the callousness dimension to be modestly correlated with internalizing problems and the uncaring dimension to be unrelated to them in a large community sample of adolescents.

Overall, our findings underscore the critical importance of assessing measurement invariance for the ICU scale across diverse and extended periods when necessary, emphasizing that this should not be taken for granted. We believe this approach is crucial for allowing meaningful comparisons between different developmental ages, avoiding misinterpretation of changes due to the characteristics of the measurement instrument as changes due to variations in the underlying construct. This could be particularly relevant, for example, when examining growth curves or developmental trajectories, where consistent measurement of the construct over time is a prerequisite.

As for some limitations of this study, we must highlight that, in this cohort, we lack some socio-environmental data (i.e., economic status, academic performance) which have been shown to be associated with callous-unemotional traits. Also, the variables were derived from self-report measures, thus a social desirability bias cannot be excluded. Future studies should address these limitations. Also, to enhance and test the generalizability and robustness of these results, further research involving samples from different countries, yet within the same age bracket, is advocated. Such cross-national replication efforts would verify whether the observed scale properties and relationships encompassing adolescence hold across varying cultural contexts. Moreover, it is essential to acknowledge the need for further investigation into certain key criterion variables to gain a more detailed understanding of the relationships among ICU dimensions. For instance, incorporating interpersonal and individual variables such as peer dynamics, family influences, and temperamental traits could offer valuable insights into potential moderators affecting adjustment outcomes.

## Summary

Firstly, this study aims to evaluate different versions of the Inventory of Callous-Unemotional (ICU) traits within a longitudinal sample of early adolescents assessed at two time points (ages 12 and 14). Results indicated that the abbreviated ICU-11 version [13], which captures the dimensions of Callousness and Uncaring, demonstrated superior data fit compared to the full 24-item scale and other shorter versions. Additionally, the study confirmed the validity of this instrument for assessing Callous and Uncaring traits consistently across male and female adolescents. Gender invariance ensures that cross-gender comparisons are meaningful, and in line with relevant literature, both traits of Callousness and Uncaring are more pronounced among males in the sample considered. Regarding longitudinal invariance, the study highlights the robust structure of the two-factor scale (Callousness and Uncaring) throughout early adolescence, confirming both configural and metric invariance; however, it fails to establish scalar invariance. This finding suggests that raw scores should not be directly compared across the considered time points without carefully addressing potential measurement artifacts, thus complicating the interpretation of longitudinal mean changes. Particularly, considering that this study examines transitions in adolescence, such shifts in item response may reflect changes in participants' interpretation rather than true changes in the underlying construct. Despite this, the presence of metric invariance of the scale allows for a valid analysis of the relationships between the Callousness and Uncaring dimensions and other relevant variables, both cross-sectionally and longitudinally. Specifically, at the cross-sectional level, the differential roles of the Callousness and Uncaring factors on target dimensions of the Strengths and Difficulties Questionnaire (SDQ) are highlighted, particularly in their relationships with prosocial behaviors, internalizing problems, and conduct disorders. The predictive value of the Callousness and Uncaring factors is discussed by observing their different longitudinal impact on SDQ dimensions at the subsequent time point, showing that their contributions differ mainly with respect to conduct problems and prosocial behaviors.

## Data Availability

Data supporting the findings of this study are available from the corresponding author, S.P., upon reasonable request.
